# Future Perspectives From a Case Report of Transcranial Magnetic Stimulation, Cognitive Behavioral Therapy, and Psychopharmacological Treatment for Post-traumatic Stress Disorder

**DOI:** 10.3389/fpsyg.2021.728130

**Published:** 2021-09-13

**Authors:** Carolina Seybert, Gonçalo Cotovio, Jaime Grácio, Albino J. Oliveira-Maia

**Affiliations:** ^1^Champalimaud Research and Clinical Centre, Champalimaud Centre for the Unknown, Lisbon, Portugal; ^2^NOVA Medical School, NMS, Universidade Nova de Lisboa, Lisbon, Portugal; ^3^Department of Psychiatry and Mental Health, Centro Hospitalar de Lisboa Ocidental, Lisbon, Portugal

**Keywords:** posttraumatic stress disorder, combined treatment, psychotherapy, psychopharmacology, treatment, transcranial magnetic stimulation

## Introduction

Post-traumatic Stress Disorder (PTSD) is a stress-related condition that occurs after experiencing or witnessing a traumatic event, such as a threat to physical integrity, or a potentially life-endangering situation, including a serious car accident, physical and/or psychological abuse, or sexual assault. It is a severe and debilitating psychiatric disorder, often resulting in professional disability, weakened relationships, decreased cognitive and psychosocial functioning, and frequent use of health care services due to physical conditions and mental comorbidities, such as substance abuse, major depressive disorder (MDD) or even suicide. The occurrence of PTSD throughout life varies between 0.56 and 6.67% among the European population (Burri and Maercker, 2014), with concerns of increasing rates due to the COVID-19 pandemic (Salehi et al., 2021). In Portugal, where the authors are based, PTSD was found to be one the most disabling mental health conditions, with patients reporting disability with six times higher likelihood than study controls (Antunes et al., 2018). Hence, there is an urge for effective and fast acting treatments in this condition. One potential successful strategy, which has become a common practice in mental health, particularly when the outcomes of first-line treatments are unsatisfactory due to inefficacy or debilitating side-effects, is combining different therapeutic modalities.

Currently available treatment options, such as psychopharmacology (Brady et al., 2000; Marshall et al., 2001) and/or psychotherapy (Monson and Shnaider, 2014; Forbes, 2020), are frequently insufficient (Hoskins et al., 2015), and innovative therapeutic strategies are necessary. In similarly debilitating disorders, such as MDD, options for therapeutic neuromodulation, such as Transcranial Magnetic Stimulation (TMS), are clinically effective (Berlim et al., 2013; Gaynes et al., 2014; Mutz et al., 2018). In fact, following approval by the US Food and Drug Administration (FDA) in 2008, TMS has been highly successful in alleviating symptoms of treatment resistant MDD and, in 2015, the UK's National Institute for Health and Care Excellence also recommended the use of TMS in MDD treatment. TMS is a non-invasive and safe neuromodulation technique, resulting from generation of an electromagnetic field by a coil which, when applied over the skull, elicits a focal electric current in the underlying cortical tissue. Such stimulation, when applied repetitively (repetitive TMS; rTMS), has more long-lasting modulatory effects on the activity of the underlying cortical nervous tissue (Radhu et al., 2016).

Over the last years, evidence has accumulated that rTMS may be effective in the treatment of PTSD (Cohen et al., 2004; Boggio et al., 2010; Watts et al., 2012; Ahmadizadeh and Rezaei, 2018), with guidelines prepared by a large group of European experts suggesting that it is probably effective (Lefaucheur et al., 2020), and supporting off-label use in selected patients. In one sham-controlled study (Ahmadizadeh and Rezaei, 2018), 58 patients with PTSD were randomized to receive 20 Hz-rTMS over the right dorsolateral prefrontal cortex (DLPFC; *n* = 19), or over the right and left DLPFC (*n* = 19), or sham stimulation (*n* = 20), for 10 sessions over 4 weeks. The parameters of stimulation for the active rTMS arms consisted in 2,400 pulses/session over the right DLPFC or 1,200 pulses over the right DLPFC followed by 1,200 pulses over the left DLPFC, performed at 100% of the resting motor threshold (RMT). A larger proportion of responders, as assessed by reduction of total score in the PTSD checklist military version, was found among the real stimulation groups, with no significant difference between unilateral and bilateral stimulation, when compared to the sham group (41.2 and 62.5 vs. 0% of responders, respectively).

Importantly, other studies have shown moderate to significant improvements when using a combination of rTMS with cognitive and behavioral interventions (Osuch et al., 2009; Isserles et al., 2013; Kozel et al., 2018). These studies combined different TMS treatment protocols with various interventions such as imaginal exposure before rTMS sessions (Osuch et al., 2009), brief script-driven exposure during deep TMS sessions (Isserles et al., 2013) and cognitive processing therapy (CPT) after rTMS sessions (Kozel et al., 2018). This is, at least in part, consistent with evidence for the therapeutic effects of psychotherapy assisted by 3,4-methylenedioxymethamphetamine (MDMA) to treat PTSD (Mitchell et al., 2021), and the use of brief symptom provocation prior to rTMS for obsessive-compulsive disorder, in accordance with the protocol cleared by the FDA (Carmi et al., 2019). Positive findings regarding the combination of rTMS and psychotherapy have also been described for other disorders, namely MDD. For example, in a large naturalistic study of 196 patients treated with at least 10 sessions of rTMS and simultaneous cognitive therapy for MDD, the authors found 66 and 56% response and remission rates, respectively, with sustained remission rates of 60% at 6 month follow up (Donse et al., 2018). Nevertheless, little is known about the neurobiological and psychological factors that might contribute toward enhancement of treatment efficacy, with further research necessary for a better understanding of such enhancement and contributing factors.

At our center, rTMS is delivered mostly for depression, but only rarely as monotherapy. In most cases, given that it is delivered due to resistance to other treatments, rTMS is applied in combination with other strategies, namely psychopharmacological treatments, but also psychotherapy. In fact, despite our current psychotherapeutic interventions not being tailored for rTMS treatment, in several patients we have observed a positive impact of rTMS on ongoing psychotherapeutic work, possibly reflecting an interaction between the two treatments. To illustrate the potential benefits of this combined intervention in PTSD, here we will present the case of a patient diagnosed with PTSD, with apparent benefit from the combination of both treatment strategies.

## Case Description

The case is that of a 45-year-old woman that, 4 years before coming to our center, had been involved in a severe car accident, while driving. After this event she reported the beginning of nightmares and daydreams involving the accident as well as disabling avoidant behaviors, such as not leaving the house due to fear of having another accident. She also reported frequent panic attacks and loss of balance without any related medical findings. She referred important relational difficulties, which culminated in separating from her husband just 1 month after the accident. Additionally, despite being highly functioning prior to the accident, 2 years after she had lost her job and since then had been incapable of resuming any working activity, due to her mental symptoms as well as chronic back pain.

Treatment for her condition had been attempted by different psychiatrists with several psychopharmacological treatments, including sertraline, trazodone, venlafaxine and quetiapine, with little or no relief. When first observed she was also taking high doses of several benzodiazepines, and attending long-term psychotherapy, as well as a pain-management program in a group setting, all without success. Concerning past psychiatric history, she had suffered a major depressive episode at ~20 years old, when her grandmother passed away. This episode was successfully treated with 2 years of psychopharmacological treatment, and it had not recurred since. The patient did not recall which specific agents were prescribed at that time. Apart from dyslipidemia, no other medical conditions were reported.

In this clinical context, she was diagnosed with PTSD and comorbid benzodiazepine use disorder. The current psychopharmacological treatment was optimized with fluoxetine, pregabalin, clorazepate dipotassium, and quetiapine. Additionally, a new psychotherapeutic treatment was offered. Due to her depressive symptoms and anxiety, the patient adhered to this treatment with difficulty, attending a total of 11 cognitive-behavioral psychotherapy (CBT) sessions over 8 months. While it was possible to discontinue almost all benzodiazepines, after 8 months of treatment with optimized doses of fluoxetine (60 mg qid), clorazepate dipotassium (30 mg qid), pregabalin (250 mg qid) and quetiapine (up to 150 mg qid), little clinical improvement was observed for PTSD symptoms, and alternative treatment options were proposed. Specifically, the patient agreed to, and signed informed consent for, off-label use of rTMS for PTSD, as an augmentation strategy for ongoing medication and psychotherapy.

## rTMS Treatment

The protocol for off-label use of rTMS for PTSD was defined according to data described in the evidence-based guidelines for therapeutic use of rTMS (Lefaucheur et al., 2020). High frequency (20 Hz) rTMS, was performed daily, Monday through Friday, in ~30 min-long sessions, with 2,400 pulses (2 s on and 28 s off) delivered at 100% of the RMT over the right DLPFC, across 6 weeks (i.e., an acute treatment cycle of 30 sessions). We used the MagVenture Coil Cool-B65, targeting the DLPFC using the “5.5 cm rule” localization method (McClintock et al., 2018). Following the acute treatment, 6 continuation sessions, performed every 2 weeks with the same stimulation protocol, were offered. During acute and continuations rTMS, the patient attended a total of 5 CBT sessions in 5 months. At the same time, the psychopharmacological treatment remained stable for fluoxetine (60 mg qid) and quetiapine SR (150 mg qid). The dose of pregabalin was gradually raised to 400 mg qid while clorazepate dipotassium (30 mg qid) was terminated.

In addition to the clinical evaluations by the treating clinicians, symptoms were assessed at the beginning, during and at the end of the rTMS treatment as presented in Table 1. The Beck Depression Inventory (BDI-II) (Beck et al., 1996; Campos and Gonçalves, 2011) was used to assess the severity of depression at baseline, weekly and at the end of the acute and continuation treatment cycles. The State Trait Anxiety Inventory (STAI) (Spielberger et al., 1983; Silva and Campos, 1998) was collected at the same time points in order to measure self-reported anxiety symptoms that commonly co-occur with PTSD (Brady et al., 2000). In order to assess PTSD symptomatology, the self-rated PTSD Checklist for DSM-5 (PCL-5) (Blevins et al., 2015; Carvalho et al., 2020), was used. Finally, at baseline and at the end of acute and continuation treatment cycles, PTSD symptoms were also assessed using Clinician-Administered PTSD Scale for DSM-5 (CAPS-5) (Weathers et al., 2018; Oliveira-Watanabe et al., 2019). After 4 weeks of the acute rTMS cycle, depressive symptom severity reduced 50% (from a BDI-II total score of 32–16) and the CAPS-5 score reduced 38% (from a total score of 47–29). A smaller reduction in symptoms severity was observed on self-reported anxiety (−11.6%; STAI-State) and PTSD symptoms (−14.5%; PCL-5). Nevertheless, an impressive symptom reduction continued until the end of rTMS continuation sessions, especially on the CAPS-5 score, that reduced almost 60% at the end of rTMS (Table 1).

**Table 1:** Clinical outcomes for combined rTMS treatment.

**Session**	**BDI-II**	**STAI-S**	**PCL-5**	**CAPS-5**
	**(% change)**	**(% change)**	**(% change)**	**(% change)**
1	32	69	76	47
30	16 (−50%)	61 (−11.6%)	65 (−14.5%)	29 (−38.3%)
36	14 (−56.3%)	55 (−20.3%)	60 (−21.1%)	19 (−59.6%)

*BDI-II, Beck Depression Inventory; STAI-S, State-Trait Anxiety Inventory State Subscale; PCL-5, Posttraumatic Stress Disorder Checklist for DSM-5; CAPS-5, Clinician-Administered PTSD Scale for DSM-5*.

In the years after completing rTMS treatment, the patient continued attending psychotherapy sessions and psychiatric appointments, albeit less frequently. Contrary to prior to rTMS, throughout the post-rTMS CBT sessions, she became able to elaborate on cognitive restructuring, as well as conduct breathing and relaxation techniques. Before rTMS treatment, she was unable to engage in any exposure techniques implemented by her cognitive-behavioral psychotherapist due to severe sadness, anguish and unbearable anxiety. For the first time, she was generalizing the use of behavioral and cognitive control strategies between sessions. Currently, the patient reports stable remission of her symptoms, as well as significant recovery of functionality.

## Discussion

In the present case, the addition of an off-label rTMS protocol for PTSD to ongoing treatments was successful. While the psychopharmacological and CBT treatments offered previously had produced unsatisfactory results, after rTMS the patient rapidly showed a decrease in symptom severity. While this may simply result from the benefit of rTMS *per se*, we believe this case report may reflect the potential impact of combining rTMS with psychotherapy. In this case, the rapid symptomatic improvement of both depressive and PTSD symptoms, once rTMS was offered, helped the patient to finally engage in psychotherapeutic work that was being offered in psychotherapy. Moreover, the well-known positive effects of rTMS on cognitive functioning (Bajbouj and Padberg, 2014) might have contributed to her deeper engagement in psychotherapy sessions. In fact, she became more prone to working with her psychotherapist and participated in techniques that had been emotionally unbearable and of limited use before starting rTMS. On the other hand, the already established psychotherapeutic relationship (Horvath et al., 2011) might have potentiated the expectation regarding TMS, prompting patient commitment to daily TMS treatment sessions, a critical moderator for clinically effective rTMS neurobiological effect (Galletly et al., 2012; Modirrousta et al., 2018). Often, for patients with PTSD, daily outgoings for TMS treatment might be challenging. This combined setting may have contributed to treatment adherence, since no rTMS session was missed for the ~5 months duration of rTMS acute and continuation treatments. Importantly, treatment adherence and expectations are known to be important contributors for better outcomes in various treatment modalities (Horwitz, 1993; Constantino et al., 2021).

Concerning the current evidence, well-illustrated by this case report, we believe that combining neuromodulation techniques with psychotherapy, as well as psychopharmacologic agents, is a promising approach in treatment of neuropsychiatric disorders, particularly in challenging or treatment-refractory cases. Nevertheless, the augmenting effect of TMS on psychotherapeutic work should be further explored, using carefully designed randomized control trials. Additionally, we believe that the neurobiological mechanisms supporting the clinical efficacy of combining these treatment modalities should also be clarified. Such path will certainly allow improving psychotherapy design as well as that of rTMS protocols. Ultimately, increasing the knowledge about combined TMS, psychopharmacological and psychotherapeutic strategies will help develop individualized treatments, increasing the likelihood of symptom remission while improving quality of life.

## Author Contributions

CS, GC, and AO-M conceived and designed the work. CS, GC, JG, and AO-M acquired and discussed the clinical case. CS and GC drafted the work. JG and AO-M revised the manuscript critically for important intellectual content. All authors approved the final version to be published and agree to be accountable for all aspects of the work in ensuring that questions related to the accuracy or integrity of any part of the work are appropriately investigated and resolved.

## Conflict of Interest

AO-M was national coordinator for Portugal of a non-interventional study (EDMS-ERI-143085581, 4.0) to characterize a Treatment-Resistant Depression Cohort in Europe, sponsored by Janssen-Cilag, Ltd (2019–2020), is recipient of a grant from Schuhfried GmBH for norming and validation of cognitive tests, and is national coordinator for Portugal of trials of psilocybin therapy for treatment-resistant depression, sponsored by Compass Pathways, Ltd (EudraCT number 2017-003288-36 and 2020-001348-25), and of esketamine for treatment-resistant depression, sponsored by Janssen-Cilag, Ltd (EudraCT NUMBER: 2019-002992-33). The remaining authors declare that the research was conducted in the absence of any commercial or financial relationships that could be construed as a potential conflict of interest.

## Publisher's Note

All claims expressed in this article are solely those of the authors and do not necessarily represent those of their affiliated organizations, or those of the publisher, the editors and the reviewers. Any product that may be evaluated in this article, or claim that may be made by its manufacturer, is not guaranteed or endorsed by the publisher.

## References

[B1] Ahmadizadeh M.-J. Rezaei M. (2018). Unilateral right and bilateral dorsolateral prefrontal cortex transcranial magnetic stimulation in treatment post-traumatic stress disorder: a randomized controlled study. Brain Res. Bull. 140, 334–340. 10.1016/j.brainresbull.2018.06.001 29883597

[B2] Antunes A. Frasquilho D. Azeredo-Lopes S. Neto D. Silva M. Cardoso G.. (2018). Disability and common mental disorders: results from the World Mental Health Survey Initiative Portugal. Eur. Psychiatry 49, 56–61. 10.1016/j.eurpsy.2017.12.004 29366849

[B3] Bajbouj M. Padberg F. (2014). A perfect match: noninvasive brain stimulation and psychotherapy. Eur. Arch. Psychiatry Clin. Neurosci. 264:27–33. 10.1007/s00406-014-0540-6 25253645

[B4] Beck A. T. Steer R. A. Ball R. Ranieri W. F. (1996). Comparison of beck depression inventories-IA and-II in psychiatric outpatients. J. Pers. Assess. 67, 588–597. 10.1207/s15327752jpa6703_13 8991972

[B5] Berlim M. T. Van den Eynde F. Jeff Daskalakis Z. (2013). Clinically meaningful efficacy and acceptability of low-frequency Repetitive Transcranial Magnetic Stimulation (rTMS) for treating primary major depression: a meta-analysis of randomized, double-blind and sham-controlled trials. Neuropsychopharmacology 38, 543–551. 10.1038/npp.2012.237 23249815 PMC3572468

[B6] Blevins C. A. Weathers F. W. Davis M. T. Witte T. K. Domino J. L. (2015). The posttraumatic stress disorder checklist for DSM-5 (PCL-5): development and initial psychometric evaluation: posttraumatic stress disorder checklist for DSM-5. J. Trauma. Stress 28, 489–498. 10.1002/jts.22059 26606250

[B7] Boggio P. S. Rocha M. Oliveira M. O. Fecteau S. Cohen R. B. Campanh,ã C.. (2010). Noninvasive brain stimulation with high-frequency and low-intensity repetitive transcranial magnetic stimulation treatment for posttraumatic stress disorder. J Clin Psychiatry. 71, 992–999. 10.4088/JCP.08m04638blu 20051219 PMC3260527

[B8] Brady K. Pearlstein T. Asnis G. M. Baker D. Rothbaum B. Sikes C. R.. (2000). Efficacy and safety of sertraline treatment of posttraumatic stress disorder: a randomized controlled trial. JAMA 283:1837. 10.1001/jama.283.14.1837 10770145

[B9] Burri A. Maercker A. (2014). Differences in prevalence rates of PTSD in various European countries explained by war exposure, other trauma and cultural value orientation. BMC Res. Notes. 7:407. 10.1186/1756-0500-7-407 24972489 PMC4114166

[B10] Campos R. C. Gonçalves B. (2011). The portuguese version of the beck depression inventory-II (BDI-II): preliminary psychometric data with two nonclinical samples. Eur. J. Psychol. Assess. 27, 258–264. 10.1027/1015-5759/a000072

[B11] Carmi L. Tendler A. Bystritsky A. Hollander E. Blumberger D. M. Daskalakis J.. (2019). Efficacy and safety of deep transcranial magnetic stimulation for obsessive-compulsive disorder: a prospective multicenter randomized double-blind placebo-controlled trial. Am. J. Psychiatry 176, 931–938. 10.1176/appi.ajp.2019.18101180 31109199

[B12] Carvalho T. Motta C. Pinto-Gouveia J. (2020). Portuguese version of the posttraumatic stress disorder checklist for DSM-5 (PCL-5): comparison of latent models and other psychometric analyses. J. Clin. Psychol. 76, 1267–1282. 10.1002/jclp.22930 31975500

[B13] Cohen H. Kaplan Z. Kotler M. Kouperman I. Moisa R. Grisaru N. (2004). Repetitive transcranial magnetic stimulation of the right dorsolateral prefrontal cortex in posttraumatic stress disorder: a double-blind, placebo-controlled study. Am. J. Psychiatry 161, 515–524. 10.1176/appi.ajp.161.3.515 14992978

[B14] Constantino M. J. Coyne A. E. Goodwin B. J. Vîsl,ă A Flückiger C. Muir H. J.. (2021). Indirect effect of patient outcome expectation on improvement through alliance quality: a meta-analysis. Psychother. Res. J. Soc. Psychother. Res. 31, 711–725. 10.1080/10503307.2020.1851058 33228466

[B15] Donse L. Padberg F. Sack A. T. Rush A. J. Arns M. (2018). Simultaneous rTMS and psychotherapy in major depressive disorder: clinical outcomes and predictors from a large naturalistic study. Brain Stimulat. 11, 337–345. 10.1016/j.brs.2017.11.004 29174304

[B16] Forbes D. (eds). (2020). Effective Treatments for PTSD: Practice Guidelines From the International Society for Traumatic Stress Studies. 3rd Edn. New York, NY: The Guilford Press.

[B17] Galletly C. Gill S. Clarke P. Burton C. Fitzgerald P. B. (2012). A randomized trial comparing repetitive transcranial magnetic stimulation given 3 days/week and 5 days/week for the treatment of major depression: is efficacy related to the duration of treatment or the number of treatments? Psychol. Med. 42, 981–988. 10.1017/S0033291711001760 21910937

[B18] Gaynes B. N. Lloyd S. W. Lux L. Gartlehner G. Hansen R. A. Brode S.. (2014). Repetitive transcranial magnetic stimulation for treatment-resistant depression: a systematic review and meta-analysis. J. Clin. Psychiatry 75, 477–489. 10.4088/JCP.13r08815 24922485

[B19] Horvath A. O. Del Re A. C. Flückiger C. Symonds D. (2011). Alliance in individual psychotherapy. Psychotherapy 48, 9–16. 10.1037/a0022186 21401269

[B20] Horwitz R. I. (1993). Adherence to treatment and health outcomes. Arch. Intern. Med. 153, 1863–8. 10.1001/archinte.1993.00410160017001 8250647

[B21] Hoskins M. Pearce J. Bethell A. Dankova L. Barbui C. Tol W. A.. (2015). Pharmacotherapy for post-traumatic stress disorder: systematic review and meta-analysis. Br. J. Psychiatry 206, 93–100. 10.1192/bjp.bp.114.148551 25644881

[B22] Isserles M. Shalev A. Y. Roth Y. Peri T. Kutz I. Zlotnick E.. (2013). Effectiveness of deep transcranial magnetic stimulation combined with a brief exposure procedure in post-traumatic stress disorder – a pilot study. Brain Stimulat. 6, 377–383. 10.1016/j.brs.2012.07.008 22921765

[B23] Kozel F. A. Motes M. A. Didehbani N. DeLaRosa B. Bass C. Schraufnagel C. D.. (2018). Repetitive TMS to augment cognitive processing therapy in combat veterans of recent conflicts with PTSD: a randomized clinical trial. J. Affect. Disord. 229, 506–514. 10.1016/j.jad.2017.12.046 29351885

[B24] Lefaucheur J.-P. Aleman A. Baeken C. Benninger D. H. Brunelin J. Di Lazzaro V.. (2020). Evidence-based guidelines on the therapeutic use of repetitive transcranial magnetic stimulation (rTMS): an update (2014–2018). Clin. Neurophysiol. 131, 474–528. 10.1016/j.clinph.2020.02.003 31901449

[B25] Marshall R. D. Beebe K. L. Oldham M. Zaninelli R. (2001). Efficacy and safety of paroxetine treatment for chronic PTSD: a fixed-dose, placebo-controlled study. Am. J. Psychiatry 158, 1982–1988. 10.1176/appi.ajp.158.12.1982 11729013

[B26] McClintock S. M. Reti I. M. Carpenter L. L. McDonald W. M. Dubin M. Taylor S. F.. (2018). Consensus recommendations for the clinical application of Repetitive Transcranial Magnetic Stimulation (rTMS) in the Treatment of Depression: (Consensus Statement). J. Clin. Psychiatry 79, 35–48. 10.4088/JCP.16cs10905 28541649 PMC5846193

[B27] Mitchell J. M. Bogenschutz M. Lilienstein A. Harrison C. Kleiman S. Parker-Guilbert K.. (2021). MDMA-assisted therapy for severe PTSD: a randomized, double-blind, placebo-controlled phase 3 study. Nat Med. 27, 1025–1033. 10.1038/s41591-021-01336-3 33972795 PMC8205851

[B28] Modirrousta M. Meek B. Wikstrom S. (2018). Efficacy of twice-daily vs once-daily sessions of repetitive transcranial magnetic stimulation in the treatment of major depressive disorder: a retrospective study. Neuropsychiatr. Dis. Treat. 14, 309–316. 10.2147/NDT.S151841 29398915 PMC5775741

[B29] Monson C. M. Shnaider P. (2014). Treating PTSD With Cognitive-Behavioral Therapies: Interventions That Work, 1st Edn. Washington, DC: American Psychological Association. 132p. 10.1037/14372-000

[B30] Mutz J. Edgcumbe D. R. Brunoni A. R. Fu C. H. Y. (2018). Efficacy and acceptability of non-invasive brain stimulation for the treatment of adult unipolar and bipolar depression: a systematic review and meta-analysis of randomised sham-controlled trials. Neurosci. Biobehav. Rev. 92, 291–303. 10.1016/j.neubiorev.2018.05.015 29763711

[B31] Oliveira-Watanabe T. T. Ramos-Lima L. F. Santos R. C. Mello M. F. Mello A. F. (2019). The clinician-administered PTSD scale (CAPS-5): adaptation to Brazilian Portuguese. Braz. J. Psychiatry. 41, 92–93. 10.1590/1516-4446-2018-0136 30758463 PMC6781712

[B32] Osuch E. A. Benson B. E. Luckenbaugh D. A. Geraci M. Post R. M. McCann U. (2009). Repetitive TMS combined with exposure therapy for PTSD: a preliminary study. J. Anxiety Disord. 23, 54–59. 10.1016/j.janxdis.2008.03.015 18455908 PMC2693184

[B33] Radhu N. Blumberger D. M. Daskalakis Z. J. (2016). “Cortical Inhibition and excitation in neuropsychiatric disorders using transcranial magnetic stimulation,” in Transcranial Direct Current Stimulation in Neuropsychiatric Disorders. eds A. Brunoni, M. Nitsche, C. Loo, editors (Cham: Springer International Publishing), 85–102. 10.1007/978-3-319-33967-2_6

[B34] Salehi M. Amanat M. Mohammadi M. Salmanian M. Rezaei N. Saghazadeh A.. (2021). The prevalence of post-traumatic stress disorder related symptoms in Coronavirus outbreaks: a systematic-review and meta-analysis. J. Affect. Disord. 282, 527–538. 10.1016/j.jad.2020.12.188 33433382 PMC7831964

[B35] Silva D. Campos R. (1998). Alguns dados normativos do Inventário de Estado- Traço de Ansiedade–Forma Y (STAI-Y) de Spielberger para a população portuguesa. Rev. Port. Psicol. 33, 71–89. 10.21631/rpp33_71

[B36] Spielberger C. D. Gorsuch R. L. Lushene R. Vagg P. R. Jacobs G. A. (1983). Manual for the State-Trait Anxiety Inventory. In Palo Alto, CA: Consulting Psychologists Press.

[B37] Watts B. V. Landon B. Groft A. Young-Xu Y. (2012). A sham controlled study of repetitive transcranial magnetic stimulation for posttraumatic stress disorder. Brain Stimulat. 5, 38–43. 10.1016/j.brs.2011.02.002 22264669

[B38] Weathers F. W. Bovin M. J. Lee D. J. Sloan D. M. Schnurr P. P. Kaloupek D. G.. (2018). The clinician-administered PTSD scale for DSM−5 (CAPS-5): development and initial psychometric evaluation in military veterans. Psychol. Assess. 30, 383–395. 10.1037/pas0000486 28493729 PMC5805662

